# Impact of bottle size on in-home consumption of sugar-sweetened beverages: a feasibility and acceptability study

**DOI:** 10.1186/s12889-017-4214-y

**Published:** 2017-04-07

**Authors:** Eleni Mantzari, Gareth J. Hollands, Rachel Pechey, Susan Jebb, Theresa M. Marteau

**Affiliations:** 1 Behaviour and Health Research Unit, University of Cambridge, Cambridge, UK; 2 Nuffield Department of Primary Care Health Sciences, University of Oxford, Oxford, UK

**Keywords:** Sugar sweetened beverages, SSBs, Consumption, Free sugars, Bottle size

## Abstract

**Background:**

Consumption of sugars-sweetened beverages (SSB) increases energy intake and the risk of obesity. Large packages increase consumption of food, implying that smaller bottle sizes may help curb SSB consumption, but there is a lack of relevant evidence relating to these products. This study explores the feasibility and acceptability of conducting a randomised controlled trial to assess the impact of different bottle sizes on SSB consumption at home.

**Methods:**

Households in Cambridge, England, which purchased at least 2 l of regular cola drinks per week, received a set amount of cola each week for four weeks, in bottles of one of four sizes (1500 ml, 1000 ml, 500 ml, or 250 ml) in random order. The total volume received consisted of a modest excess of households’ typical weekly purchasing, but was further increased for half the study households to avoid ceiling effects. Consumption was measured by recording the number of empty bottles at the end of each week. Eligible households were invited to complete a run-in period to assess levels of active participation.

**Results:**

Thirty-seven of 111 eligible households with an interest in the study completed the run-in period. The study procedures proved feasible. The target for recruitment (*n* = 16 households) was exceeded. Measuring consumption was feasible: over three quarters (*n* = 30/37) of households returned all bottles on the majority (*n* = 88/101) of the study weeks completed across households. The validity of this measure was compromised by guests from outside the household who drank the study cola (*n* = 18/37 households on 48/101 study weeks) and consumption of the study cola outside the home. Supplying enhanced volumes of cola to nine households was associated with higher consumption (11,592 ml vs 7869 ml). The intervention and study procedures were considered acceptable. Thirteen households correctly identified the study aims.

**Conclusion:**

The findings support the feasibility and acceptability of running a randomised controlled trial to assess the impact of presenting a fixed volume of SSB in different bottle sizes on in-home consumption. However, methods that avoid consumption being influenced by the amount of cola supplied weekly by the study and that capture out of home consumption are needed before conducting a randomised controlled trial.

**Trial registration:**

ISRCTN14964130; Registered on 18th May, 2015.

## Background

Intake of free sugars in the population exceeds recommendations, with the largest source in the diet being sugars-sweetened beverages (SSBs) [1, 2]. SSB consumption increases energy intake and the risk of obesity [3–5], is linked to adverse health consequences [6–8] and contributes to health inequalities, given greater consumption amongst the most deprived households [9–12]. A recent Cochrane systematic review found that exposure to large portions and packages increases the consumption of food and non-alcoholic drinks [13], implying that smaller bottle sizes may help to curb consumption of SSBs. The importance of developing interventions and policies to reduce the size, availability, and appeal of large portions was also highlighted in a recent analysis [14]. As most of the studies included in the aforementioned review targeted food products and compared standard vs. larger packages rather than standard vs small packages, the impact of small bottles on SSB consumption is unclear. To address the absence of relevant evidence, we are planning to conduct a crossover randomised controlled trial to assess the impact of presenting a fixed volume of sugar-sweetened beverages in different bottle sizes on consumption in homes. Prior to conducting this trial, however, there is a need to reduce key uncertainties related to its design. The aim of the current study, therefore, is to assess the feasibility and acceptability of the procedures for recruitment, allocation, measurement, retention and intervention delivery of the aforementioned randomised controlled trial.

## Methods

The study design and methods have been previously published [15]. In brief, the study used a crossover design in which residential households received a set amount of cola each week for four weeks, based on their typical weekly purchasing, in bottles of one of four sizes: 1500 ml, 1000 ml, 500 ml, or 250 ml, in random order. The unit of randomisation was the household. Each intervention period lasted one week. The amount received by each household was determined by till receipts collected during a two-week baseline period.

Eligible households, i.e. those which purchased at least 2 l of regular cola drinks (Coca Cola or Pepsi Cola) per week and lived in Cambridgeshire England, were identified and  recruited through a research agency. In line with the characteristics of the population of SSB consumers in the UK [10], we aimed for 50% of the recruited households to be from areas of high deprivation, as defined by their Index of Multiple Deprivation (IMD) [16] score, with areas falling under the fourth and fifth IMD quintile considered highly deprived. One individual from each eligible household was recruited to act as a household representative, who consented to participation in the study for the entire household and provided all necessary data.

In order to evaluate recruitment rates, 110 eligible households were initially approached to assess how many would be interested in taking part in the study, inferred by completion of a one-week run-in period, serving to acclimatise households to the range of different SSB bottle sizes. Of the households completing the run-in period and expressing a willingness to continue with the study (*n* = 37), 16 were randomly selected (using a random number generator) to be invited to continue participation. Seven of these received their typical weekly amount of cola rounded up to the nearest multiple of three litres and the remaining nine were supplied with an additional three litres, to avoid ceiling effects (i.e. consistent consumption of all provided cola, regardless of bottle size) that were observed in the consumption of two out of the seven first households which completed the intervention periods.

Households were not fully informed at recruitment of the study’s aim, as it was assumed that such knowledge might differentially affect consumption with each bottle size. Instead, household representatives were told that the study involved a consumer research exercise, aiming to determine whether and how different bottles affect people’s consumption experiences, including perceptions of taste, level of enjoyment and satisfaction associated with beverage consumption, perceived product quality,purchasing likelihood, and attitudes towards different bottles, including their appeal and user-friendliness. To build credibility for the cover story, at the end of each intervention period, household representatives were asked to rate their consumption experiences, as well as the experience of any visitors they might have had, who had consumed part of their SSB stock. In order to determine whether households believed the cover story or were aware of the purpose of the intervention and of the study’s aim, each household representative was asked at the final follow-up assessment what they thought the study was about.

Consumption was assessed each week by recording the numbers of empty bottles, which households were requested to retain, and measuring the volume in remaining full and partially full bottles. Bottles were collected at the end of each intervention week by a member of the research team, who also provided the cola for the following week. Visiting times were kept fixed each week for each household, to ensure that each intervention period was of equal duration and to avoid any unintended impacts on consumption. Other outcomes recorded included recruitment and loss-to-follow-up rates and socio-demographic characteristics of participating households. The acceptability of the intervention and study procedures was assessed in interviews conducted at the end of the intervention periods, during which household representatives were questioned about their experiences of taking part in the study, including their experiences of consuming cola from the different bottle sizes. During the interviews, awareness of the study’s aim was also explored. The procedures and findings of this qualitative component are described elsewhere (Mantzari E, Hollands GJ, Pechey R, Jebb S & Marteau TMM: Perceived impact of smaller vs large-sized bottles of sugar-sweetened beverages on consumption: a qualitative analysis, in submission).

At the end of the study, household representatives were fully debriefed on the study aims. Each household received £150 worth of shopping vouchers for completion of all intervention periods and follow-up assessments. Households completing the run-in period but not invited to continue their participation, or not interested in continuing, received £30 worth of shopping vouchers.

## Results

### Feasibility of recruiting and retaining eligible participants

**Fig. 1:**
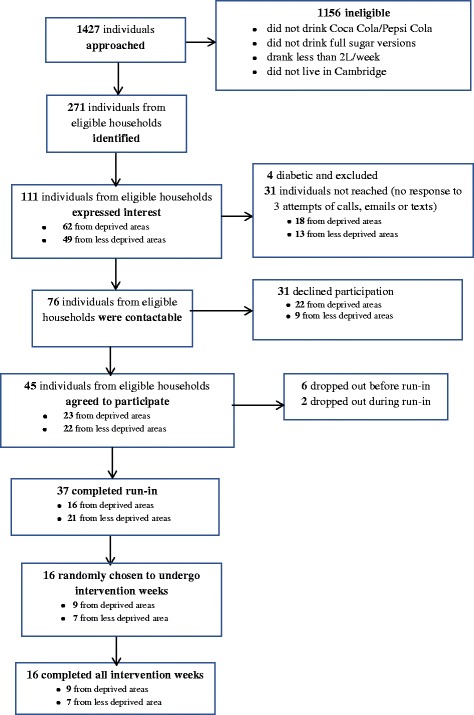
Flow of participants through study

Of the 1427 individuals approached, 271 (19%) were from eligible households, of whom 111 (41%) expressed an interest in the study, 45 (28%) agreed to take part and 37 (13%) completed the run-in phase, i.e. the first week of the study, which was considered an index of active participation. As per protocol, 16 households (6% of those eligible; 14% of the 37 that completed the run-in period) were randomly selected (using a random number generator) to undergo the intervention. Attrition between consenting to take part and completion of the run-in was 18% (8/45). No households dropped out between completion of the run-in phase and the first intervention period or between the four intervention periods (Fig. 1).

The majority (79%: 29/37) of recruited households consisted of families with children and had a mean of 3.5 members (sd = 1.3; range 1–6). The mean number of children per household was 1.7 (sd = 1.2.; range: 0–4) with a mean age of 8 years (sd = 3.9). The mean adult age was 36 years (sd = 9.9). Approximately half of all household members were male (51%). The educational level (as assessed by the highest educational qualification received by anyone in a household) of the majority of recruited households was classified as higher (i.e. beyond A levels or equivalent) (54%: 20/37) but their annual income was classified as low (up to £25 K) (62%: 23/37). Based on area level deprivation (Index of Multiple Deprivation scores) 43% (16/37) of households were classified as deprived.

### Feasibility of study procedures

No major problems were reported with the study procedures, including delivery of the intervention. Some minor issues were as follows:

Nine households (24% of the 37 that completed the run-in), four of whom were randomised to complete the intervention periods (25% of the 16 randomised), provided till receipts for only one of the two baseline weeks used to determine typical weekly purchasing.

Four households (11% of the 37 that completed the run-in), rescheduled their appointments on certain intervention weeks, resulting in some variation in appointment times, but only by a few hours. A further six households (16% of the 37 that completed the run-in) made appointment-time changes that affected the duration of their run-in period.

Two households (5% of the 37 that completed the run-in), who were randomised to complete the run-in only, self-reported purchasing additional cola for in-home consumption rather that requesting additional deliveries.

Thirty-five percent of 37 households, five of whom were randomised to complete the intervention weeks (31% of the 16 randomised) guessed the study aim but did not guess the expected direction of effect. When interviewed about their experiences of taking part in the study, those who guessed the aim reported that this knowledge did not affect their consumption with each bottle size. Those who did not identify the aim also reported that having such knowledge would not have affected their behaviour towards each bottle size.

### Feasibility of measuring consumption

Seven households (19% of the 37 households that completed the run-in) failed to return all their bottles on certain weeks. Of the 101 study weeks completed across households, missing bottles were reported during 13 weeks (13% of overall study weeks). However, household representatives confirmed that missing bottles were empty, allowing estimation of the amount consumed by subtracting leftover amounts from the total volume delivered.

Eighteen households (49% of the 37 households that completed the run-in) self-reported having guests who drank a proportion of their cola on 48 of the 101 study weeks. Some households reported using some bottles for out-of-home consumption, with uncertain effects on overall consumption in the absence of an overarching measure of intake.

Mean consumption across households with each bottle size (i.e. observed during each one-week intervention period) can be seen in Table 1. Mean overall consumption across interventions periods (i.e. across all 4-week intervention periods, regardless of bottle size) was 7869 ml (sd = 1289) for the seven households that received a modest excess of their typical weekly cola amount and 11,592 ml (sd = 41,203) for the nine households that were oversupplied.

**Table 1:** Consumption (ml) across households (*n* = 16) with each bottle size (each used for one week)

Bottle size	Mean (sd)
1.5 L	8010 (sd = 3977)
1 L	8331 (sd = 3963)
500 ml	8595 (sd = 3559)
250 ml	7878 (sd = 3861)

### Acceptability to participants

The study procedures, assessments and intervention were considered acceptable and no problems were reported by participating households. Participants expressed positive attitudes towards the study aims and procedures (“*brilliant study…. I found it all easy”* (Household 3)); *“the study was interesting”* (Household 7); *“interesting and quite easy to do”* (Household 42)) and described the convenience of having the drinks delivered to them (“*that was easier (getting the cola delivered) for me because it meant I didn’t have to keep worrying about it”* (Household 37)). No issues were reported with removing existing drinks prior to the start of the intervention periods, as these were compensated for and replaced (“*she replaced it so it’s not like she’s just robbing us of drink”* (Household 5)). The use of a cover story was met with understanding and did not evoke any negative responses (*“no, it doesn’t worry me, because obviously there’s a very good reason why you didn’t tell us…because if you told us then you wouldn’t have had a true reflection of the study and then there would’ve been a waste of time”* (Household 2)).

## Discussion

This study assessed the feasibility and acceptability of presenting a fixed volume of SSB in different bottle sizes as a possible intervention for reducing in-home consumption. The study provided evidence for the feasibility of identifying, recruiting and retaining eligible households: the target sample of 16 households was successfully met by approaching the pre-specified number of 100 eligible households and approximately half of the recruited households were from deprived areas (i.e. from areas falling under the 4th and 5th IMD quintile), thus matching the deprivation level of typical SSB consumers in the UK [10]. Furthermore, attrition rates were low with all households who were randomised to undergo the intervention periods completing the study. The study also supports the feasibility of the study procedures: no major issues were reported with running the study, including delivering the intervention. The majority of participants were not aware of the study’s scientific aim, demonstrating the relative success of the cover story. Furthermore, results support the acceptability to participants of the study procedures, assessments and intervention.

Consumption was assessed by recording the numbers of empty and remaining full bottles, a measure found to be feasible in the present study: most households adhered to the instructions to keep all bottles, regardless of whether the contents were full, partially full or empty, thus allowing estimation of consumption from leftover amounts in bottles. This was most likely facilitated by the fact that households were requested to pay for the SSB they consumed and the rate was determined by the amount they had consumed in the previous week. However, the validity of assessing consumption from empty bottles was compromised by the drinking of the household cola by guests. The presence of guests would be expected to be randomly distributed across groups in a randomised controlled trial, thus eliminating the threat of this issue to the validity of the measure. Nonetheless, a potential method of overcoming this in future studies would be to instruct households to refrain from sharing their cola with guests.

A further factor undermining the validity of the measure of consumption was the failure to capture out-of-home consumption. The different bottle sizes differed in their portability, and thus in their likelihood of being used for out-of-home consumption. Interviews conducted with study households [reported elsewhere (Mantzari E, Hollands GJ, Pechey R, Jebb S & Marteau TMM: Perceived impact of smaller vs large-sized bottles of sugar-sweetened beverages on consumption: a qualitative analysis, in submission)] suggest that compared to larger bottles, smaller bottles were more likely to be taken out of the house (e.g. at work, while shopping etc), thus minimising the possibility of additional cola being purchased out of the home and possibly increasing consumption through having cola readily available in that context. Measured consumption levels with smaller bottles, therefore, might reflect total consumption (i.e. in- and out-of-home), while consumption with larger bottles might reflect true in-home consumption. To capture both in- and out-of-home consumption, future studies should incorporate additional measures to empty bottle count, including till receipts and self-report.

The study was not powered to detect differences in consumption with the difference bottle sizes. Due to the change in protocol, however, half-way though the study regarding the amount delivered, it was possible to observe that regardless of bottle size, consumption levels reflected study-determined supply. Ceiling effects were observed when supplying households with a modest excess of their usual weekly cola amounts. Anecdotal evidence suggests that supplies lasted only a few days, as opposed to in excess of a week, implying an increased speed of consumption. Supplying enhanced volumes of cola to avoid such effects was associated with higher consumption. This suggests that household availability of SSB is an important determinant of consumption, with stockpiling of bottles unintentionally changing consumption-related behaviours. One possible way of overcoming this would be to reduce the amount of cola available within the home at any one time, by supplying it at more frequent intervals, for example on a bi-weekly rather than on a weekly basis. It would still be challenging, however, to ensure that the change in the supply of cola relative to habitual purchasing patterns does not impact on consumption patterns.

## Conclusion

In conclusion, the findings of this study support the feasibility and acceptability of running a randomised controlled trial to assess the impact of presenting a fixed volume of SSB in different bottle sizes on in-home consumption. Issues, however, were identified associated with the study design, which are likely to impact on the validity of the primary outcome, i.e. consumption level. These include consumption driven by study-determined supply and a failure to capture out-of-home consumption. Methods to avoid these would be needed before conducting a definitive trial.
